# A Phase II Study Investigating Cabozantinib in Patients with Refractory Metastatic Colorectal Cancer (AGICC 17CRC01)

**DOI:** 10.1158/2767-9764.CRC-22-0169

**Published:** 2022-10-14

**Authors:** Aaron J. Scott, Atrayee Basu Mallick, Efrat Dotan, Steven J. Cohen, Philip J. Gold, Howard S. Hochster, Somasundaram Subramaniam, Afsaneh Barzi, George S. Watts, Patrick J. Blatchford, Wells A. Messersmith

**Affiliations:** 1 Division of Hematology and Oncology, University of Arizona Cancer Center, Tucson, Arizona.; 2 Sidney Kimmel Cancer Center, Thomas Jefferson University, Philadelphia, Pennsylvania.; 3 Fox Chase Cancer Center, Philadelphia, Pennsylvania.; 4 Jefferson Health/Abington Hospital, Sidney Kimmel Cancer Center, Philadelphia, Pennsylvania.; 5 Swedish Cancer Institute, Seattle, Washington.; 6 Rutgers Cancer Institute of New Jersey, New Brunswick, New Jersey.; 7 City of Hope Comprehensive Cancer Center, Duarte, California.; 8 Colorado School of Public Health, Aurora, Colorado.; 9 University of Colorado Cancer Center, Aurora, Colorado.

## Abstract

**Purpose:**

Multi-tyrosine kinase inhibitors (TKI) have shown clinical activity in patients with metastatic colorectal cancer. Cabozantinib, a multi-TKI, exhibited potent antitumor activity superior to regorafenib in preclinical colorectal cancer patient-derived tumor xenograft models. This phase II study aimed to investigate cabozantinib, a multi-TKI, in patients with refractory, metastatic colorectal cancer (mCRC).

**Experimental Design:**

A nonrandomized, two-stage, phase II clinical trial evaluating 12-week progression-free survival (PFS) was conducted in eight cancer centers across the United States between May 2018 and July 2020.

**Results:**

A total of 44 patients were enrolled between May 2018 and May 2019, 40 of which were response evaluable. Of the total 769 reported adverse events (AE), 93 (12%) were ≥ grade 3. Five grade 5 AEs were reported of which four were unrelated to study drug and one was reported as possibly related due to bowel perforation. Eighteen patients (45%) achieved 12-week PFS with stable disease or better (confidence interval, 0.29–0.62; *P* < 0.001). One patient (3%) had a partial response, and 27 other patients achieved stable disease as best response per RECISTv1.1. Median PFS was 3.0 months, and median overall survival was 8.3 months. Of the 18 patients who achieved 12-week PFS, 12 had left-sided primary tumors, 11 were *RAS* wild type, 11 were *PIK3CA* wild type, and 6 had previous regorafenib therapy. The 12-week PFS rate was higher in *RAS* wild-type tumors compared with *RAS* mutant tumors (0.61 vs. 0.32; *P* = 0.11).

**Conclusions:**

This phase II study demonstrated clinical activity of cabozantinib in heavily pretreated, patients with refractory mCRC, and supports further investigation.

**Significance:**

Targeting angiogenesis through VEGF axis blockade provides incremental survival benefit in patients with mCRC. The hepatocyte growth factor/MET signal transduction pathway has been observed as a mechanism for acquired resistance. Dual inhibition of VEGF plus MET is an attractive therapeutic strategy. This phase II trial demonstrated clinical activity with cabozantinib, a multi-TKI targeting VEGFR2 and MET, in patients with refractory, mCRC.

## Introduction

Colorectal cancer is the fourth most commonly diagnosed and second most deadly cancer in the United States (1). While several treatment options for metastatic colorectal cancer (mCRC) have led to improved survival and response rates over the past several decades, prognosis remains poor with an estimated median overall survival (OS) of 30 months and a 5-year OS rate of 14% (1). Standard-of-care agents in microsatellite stable (MSS) mCRC include first-line chemotherapy using sequential or in combination fluoropyrimidines, oxaliplatin, and irinotecan, and, depending on *RAS* and *BRAF* mutation status, mAbs targeting EGFR (cetuximab and panitumumab) and antiangiogenic therapy targeting the VEGF axis (bevacizumab: VEGFR; ramucirumab: VEGF-trap; ziv-aflibercept; ref. 2). On the basis of the phase III BEACON trial, encorafenib, an oral small-molecule BRAF inhibitor, plus cetuximab has been FDA approved for treatment of subsequent line, *BRAF*-mutant mCRC (3). mAbs targeting immune checkpoint proteins (i.e., PD-1, nivolumab, pembrolizumab; and CTLA-4, ipilimumab) have also been FDA approved for use as first-line agent for microsatellite instable (MSI) tumor types (4, 5). Clinically relevant biomarkers for treatment of mCRC also include *HER2/Neu* overexpression and *NTRK* fusions (6). Recently, studies have also shown potential for targeted inhibitors eliciting clinical responses in *KRAS* G12C-mutant colorectal cancer (7, 8). Regorafenib received FDA approval for treatment of metastatic, refractory colorectal cancer in 2015 based on the phase III “Regorafenib monotherapy for previously treated metastatic colorectal cancer (CORRECT) trial”, although regorafenib offers modest survival benefit of approximately 1.4 months over best supportive care and treatment resistance is inevitable (9, 10). TAS-102, an orally administered combination of trifluridine plus tipiracil hydrochloride, has also been FDA approved for marginal survival benefit in patients with refractory, mCRC. As more biomarker-driven studies have shown improved outcomes for a variety of molecular targets, the landscape for treating mCRC has become more complex as patients progress through therapeutic regimens. Although therapeutic options have led to improved survival, many patients ultimately progress on all standard-of-care agents regardless of tumor biology and targeted therapy, highlighting the need for ongoing research and novel therapies for mCRC.

Therapeutic resistance, both inherent and acquired, has been well described as an important reason for poor outcomes in mCRC. Angiogenesis has been well described as a hallmark for colorectal cancer tumorigenesis. Specifically, VEGF ligand binding to VEGFR2 has been shown as a driver of tumor neovascularization (11). While antiangiogenics have demonstrated incremental improvement in survival benefit, resistance to angiogenic inhibition uniformly develops over months in patients with mCRC (12, 13). One of the mechanisms by which acquired resistance occurs is through upregulation of the proto-oncogene mesenchymal–epithelial transition (cMET) receptor tyrosine kinase (RTK; ref. 14). Upon binding to its ligand hepatocyte growth factor (HGF), the cMET signaling pathway controls tumor cell survival and metastasis in part via activation of the PIK3CA/AKT/mTOR pathway as well as contributing to antiangiogenic therapeutic resistance via upregulation of *MET* expression (15–17).

Previous reports have observed cMET overexpression in 30%–75% and *MET* amplification in 3%–4% of patients with mCRC analyzed by IHC and PCR, respectively (18). Targeting the cMET/HGF axis may restore efficacy of the antitumor effects of antiangiogenesis, which has been previously reported by our group investigating cabozantinib (XL184), a multi-tyrosine kinase inhibitor (TKI) targeting VEGF and MET, in patient-derived xenograft (PDX) mouse models (19). However, the clinical utilization of cMET overexpression/*MET* amplification as a predictive biomarker in colorectal cancer is unproven and limitations such as a lack of consensus regarding method for analysis of expression/amplification and definition of positive values remain challenges for clinical interpretation.

Cabozantinib is an orally administered, RTK inhibitor of multiple kinases effecting cancer cell growth, angiogenesis, and metabolism including MET, VEGFR2/KDR, AXL, TIE2, RET, and KIT. Cabozantinib has demonstrated clinical effectiveness in a variety of tumor types with NCCN guideline recommendations for use in metastatic renal cell carcinoma, metastatic/unresectable hepatocellular carcinoma, metastatic medullary thyroid cancer and imatinib-resistant gastrointestinal stromal tumors (20–23). Similarities among mechanism of action between other RTK inhibitors and cabozantinib exist including regorafenib, which also targets VEGFR2; however, cabozantinib provides the unique inhibitory profile of dual inhibition of VEGFR2 and MET.

Preclinically, cabozantinib has shown impressive antitumor activity in PDX mouse models with tumor growth inhibition in 80% of treated colorectal explants (24). Moreover, cabozantinib also demonstrated superior tumor growth inhibition, reduction in tumor vascularity, and increase in autophagy compared to regorafenib in colorectal PDX mouse models (19). On the basis of these data, we designed a phase II Simon two-stage, nonrandomized trial aimed to investigate the efficacy and safety of cabozantinib in metastatic, refractory colorectal cancer (NCT03542877).

## Material and Methods

### Patients

This prospective study was performed through eight academic centers (members of the “Academic GI Cancer Consortium” or AGICC) in the United States and enrolled patients with histologically proven refractory mCRC between May 2018 and May 2019. The data cut-off date was August 4, 2021. Patients must have progressed on or were intolerant to fluoropyrimidine, irinotecan, oxaliplatin, and bevacizumab, and prior EGF inhibitor therapy was required for patients with left-sided, *RAS* wild-type (WT) tumors. Prior regorafenib or TAS-102 treatment was allowed but not required. Patients must have had an Eastern Cooperative Oncology Group (ECOG) performance status of 0 or 1 and had measurable disease per RECIST, version 1.1 (v1.1) as determined by the investigator. Patients with evidence of cavitating lung lesions, tumors invading or encasing major blood vessels, or demonstrating clinically significant gastrointestinal bleeding within 6 months before treatment start were excluded from the trial. Patients requiring concomitant anticoagulation at therapeutic doses with oral anticoagulants or platelet inhibitors were also excluded from the trial. Cabozantinib was administered at a starting dose of 60 mg orally daily based on previously established safety and efficacy data in other tumor types (25, 26). Two dose-level reductions were allowed per protocol (40 and 20 mg). Response-evaluable patients were those patients who had radiographic and/or clinical assessments to evaluate for disease progression or response per RECIST v1.1.

Investigators obtained written informed consent from the patients, procedures related to this trial were conducted in accordance with recognized ethical guidelines, and this study was approved by an Institutional Review Board.

### Circulating Tumor DNA Analysis

Whole blood for circulating tumor DNA (ctDNA) analysis was collected at baseline and on treatment ± 5 days of cycle 3 day 1 from consenting patients to test the correlation of ctDNA response to clinical response. Whole blood draws (∼8 mL) were collected and processed to plasma by centrifugation at 1,600 × *g* for 10 minutes followed by a second spin at 16,000 × *g* for 10 minutes. Plasma was collected and frozen at −80°C. Two milliliters of plasma was used to isolate cell-free DNA (cfDNA) with the Qiagen circulating nucleic acid isolation kit (#55114) according to manufacturer's protocols. Following quantitation of the cfDNA using a Qubit fluorimeter, 10 ng of cfDNA was used to generate sequencing libraries with the Ion Torrent, Ion AmpliSeq Library kit and Ion AmpliSeq Cancer Hotspot V2 primer panel according to the manufacturer's protocols. Libraries were sequenced on either the Ion Torrent Proton P1 chip or the Ion Torrent Personal Genome machine 318 chip with a minimum average coverage of >1,200×. Variants were called using Ion Reporter software (v5.12) and filtered for known pathogenic and/or likely pathogenic mutations.

### Statistical Analysis

#### Sample Size Determination

The primary endpoint for this trial was the binary endpoint of being progression free at 12 weeks. On the basis of the phase III CORRECT trial investigating regorafenib versus best supportive care, the median progression-free survival (PFS) for patients with refractory colorectal cancer was 1.7 months in the placebo arm. Therefore, the PFS at 12 weeks would be approximately 13%, which served as the historical control for the null hypothesis. This study was designed to have at least 90% power to detect an improvement in the 12-week PFS rate of at least 20%; therefore, the alternative hypothesis is that the 12-week PFS rate is 33%. The optimal design providing at least 90% power to detect the alternative while controlling the type I error rate at 0.05 uses at most 44 patients, thus 44 patients were enrolled to this trial. Using an optimal Simon two-stage design, the first 16 patients were enrolled and 12-week PFS was determined. An additional 28 patients were enrolled after meeting the predetermined criteria for trial continuation, resulting in a total of 44 patients enrolled. With this design, the therapy would be considered efficacious if 10 or more patients were free of progression at 12 weeks, suggesting that the treatment has sufficient efficacy to warrant future study.

#### Survival and Response Analysis

Disease assessment was performed by CT or MRI methods at baseline and every 6 weeks for the first 12 weeks, then every 9 weeks thereafter during treatment until disease progression. Response evaluation was performed on the basis of RECIST v1.1. PFS was defined as the time from administration of the initial dose of cabozantinib to evidence of radiographic progression as defined by RECIST or death from any cause without evidence of disease progression, whichever occurred first. Kaplan–Meier estimates of PFS and OS rates were calculated along with their corresponding 95% confidence intervals. Objective response rate (ORR) was defined using the RECIST v 1.1 as the proportion of patients with a confirmed partial response (PR) or complete response. OS was defined as the time from administration of the initial dose of cabozantinib until death from any cause. Surviving patients who were taken off trial prior to clinical or radiographic disease progression were censored from primary endpoint analysis.

#### Biomarker Analysis

Historic results of tissue-based molecular profiling were retrieved on all enrolled patients. Retrospective analysis of PFS and response rate (RR) in patients based on *RAS*, *BRAF*, and *PIK3CA* mutation status was performed using point estimates with 95% confidence intervals and comparisons were made using a stratified log-rank test. Carcinoembryonic antigen serum levels were performed at baseline and every 3 weeks during treatment.

#### Safety Analysis

Safety and tolerability analysis of cabozantinib was summarized by dose and severity as assessed by the Common Toxicity Criteria for Adverse Events version 4.0 and relationship to study drug.

#### ctDNA Analysis

ctDNA correlation to clinical response was performed in patients who had pretreatment and posttreatment blood samples obtained, and in whom the mutation prevalence changed by ≥ 20% following therapy. Patients were grouped into those whose disease progressed or those whose disease remained stable or responded to therapy. A one-tail Fisher exact test was performed to determine whether there was a statistically significant association between the change in mutational prevalence and clinical response.

### Data Availability

The human sequence data generated in this study are not publicly available due to patient privacy requirements but are available upon reasonable request from the corresponding author. Other data generated in this study are available within the article and its Supplementary Data files.

## Results

### Characteristics of Patients

**FIGURE 1:**
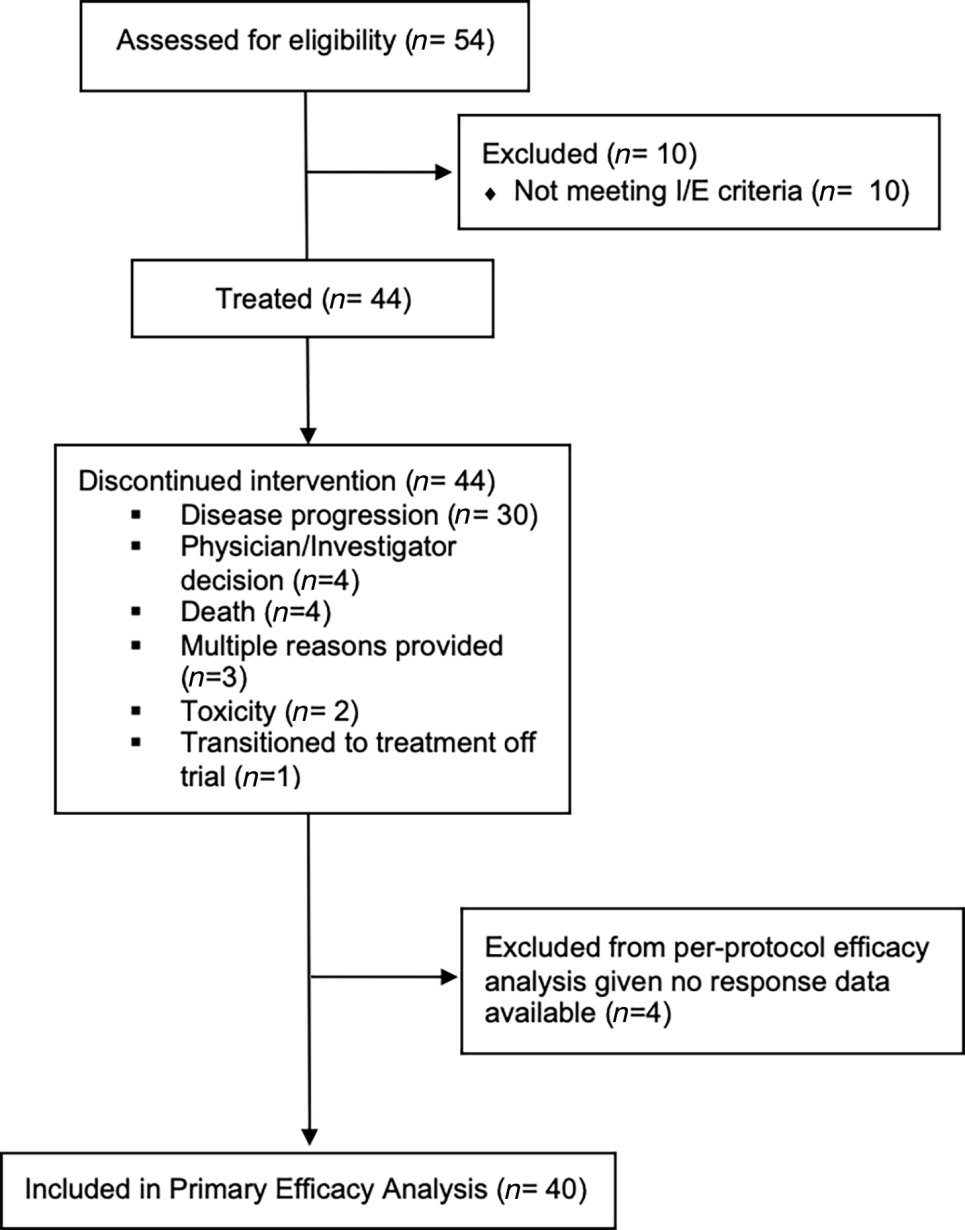
CONSORT diagram.

A total of 44 patients with mCRC were included in this study (male, 57%; median age, 60 years; Table 1). Of the 44 patients enrolled, 40 patients were response evaluable based on prespecified definition for primary endpoint analysis. The study met the predetermined criteria for expansion and total planned patient enrollment based on the Simon two-stage design. The median number of previous lines of therapy was 4. The median time from metastatic diagnosis to informed consent on this trial was 30.7 months. Eight (18%) patients had previous regorafenib treatment. *KRAS* mutations were present in 54% of patients. No additional clinically relevant molecular aberrations were reported including *NRAS* mutations, *BRAF* mutations, *HER2* overexpression, *NTRK* fusions, or MSI-high (MSI-H) tumor types. Other patient characteristics and treatment course are summarized in Table 1 and Figure 1.

**TABLE 1:** Patient characteristics (all patients treated; *N* = 44)

Characteristic:	*n* (%)
Median age (minimum to maximum)	60 (38–75)
Sex	
Men	25 (57%)
Women	19 (43%)
Race	
Caucasian	37 (84%)
Black	3 (7%)
Asian	1 (2%)
Other (not specified)	3 (7%)
ECOG at screening	
0	21 (48%)
1	23 (52%)
Primary site of disease	
Left sided	29 (66%)
Right sided	13 (30%)
Transverse	1 (2%)
Not specified	1 (2%)
KRAS mutation^a^	
Yes	24 (54%)
No	20 (46%)
PIK3CA mutation^b^	
Yes	7 (16%)
No	24 (54%)
Unknown	13 (30%)
Number of previous systemic anticancer therapies (metastatic setting)	
2	7 (16%)
3	11 (25%)
≥4	26 (59%)
Type of previous systemic anticancer therapies	
Oxaliplatin-based	44 (100%)
Irinotecan-based	43 (98%)
Anti-VEGF	43 (98%)
Anti-EGFR	14 (32%)
Regorafenib	8 (18%)
TAS-102	11 (25%)
Time from diagnosis of metastasis	
Median (months)	30.7
<18 months	12 (27%)
≥18 months	32 (73%)

NOTE: No patients harbored *BRAF* mutant or MSI tumors.

^a^KRAS exons 2, 3, and 4.

^b^PIK3CA exons 9, 10, and 20.

### Survival and Response

**FIGURE 2:**
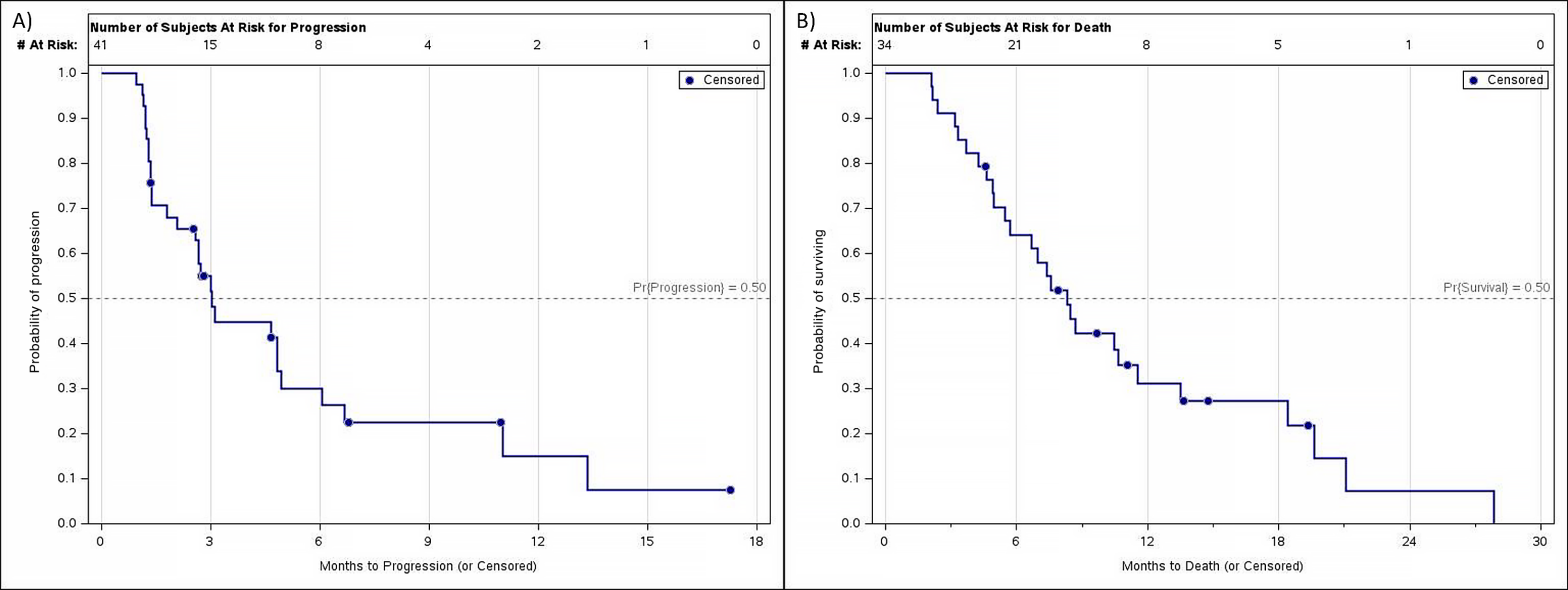
Kaplan–Meier curves for PFS (**A**) and OS (**B**).

**FIGURE 3:**
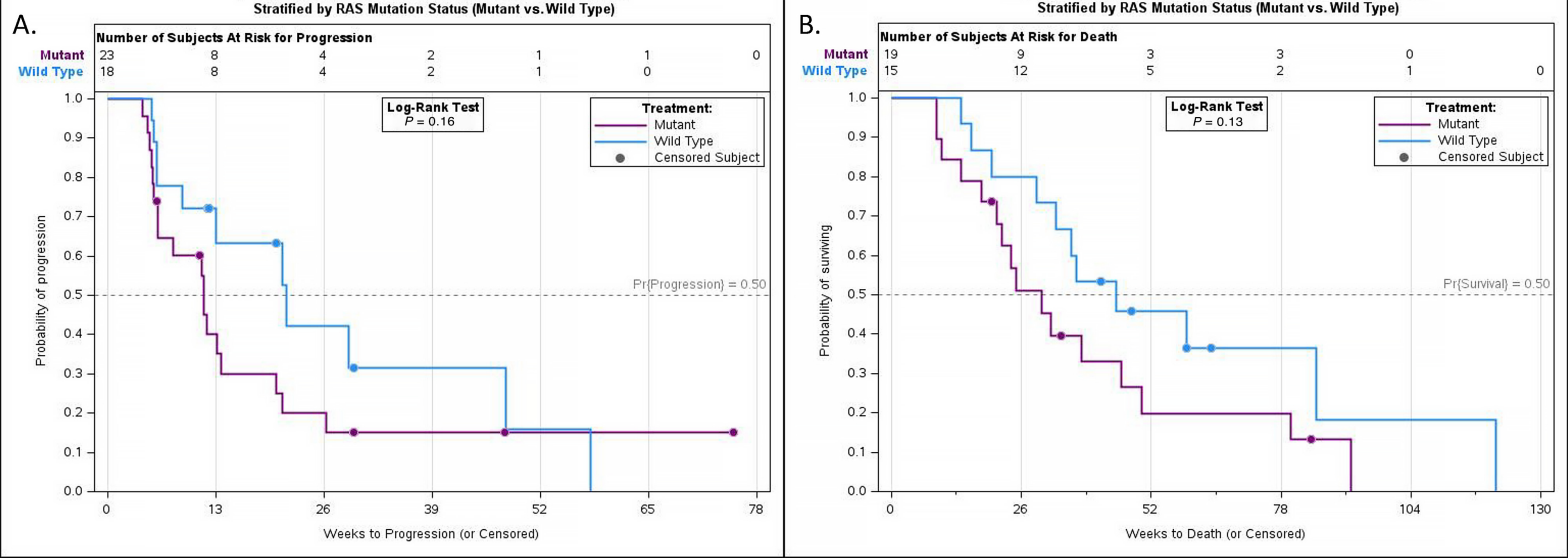
Kaplan–Meier analysis in response-evaluable patients for PFS (**A**) and OS (**B**) stratified by *RAS* mutation.

**FIGURE 4:**
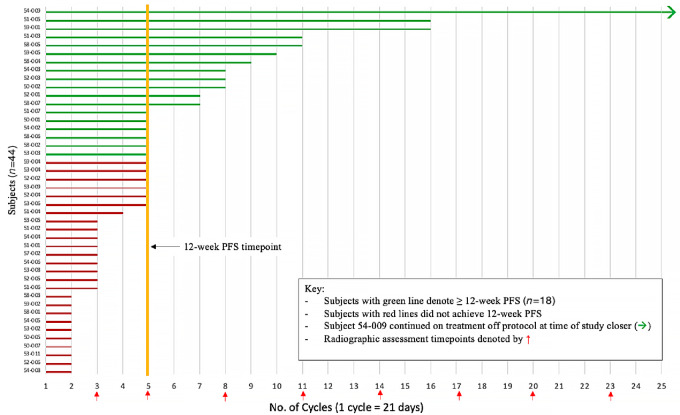
Swimmers plot for duration on treatment for total subjects (*n* = 44).

Among the 40 response-evaluable patients, 18 (45%) patients were free of progression at 12 weeks. Three patients discontinued treatment prior to disease assessment. The median PFS was 3.0 months, and the median OS was 8.3 months (Fig. 2). A trend toward improved PFS in patients with *RAS* WT versus mutant (MT) mCRC was observed (4.9 vs. 2.7 months, respectively; *P* = 0.16; Fig. 3). A trend toward improved OS in patients with *RAS* WT versus MT tumors was also observed (10.4 vs. 7.0 months, respectively; *P* = 0.13; Fig. 3). No clinically or statistically significant differences in PFS or OS were observed in the *PIK3CA* MT versus WT subgroup analysis (Supplementary Fig. S1). One patient demonstrated a PR and 27 other patients had stable disease (SD) as their best response, giving an ORR of 2.5% and a disease control rate of 70% (Supplementary Fig. S2). One patient maintained a PR through trial closure and was continued on cabozantinib through compassionate use at last follow-up (Fig. 4). Follow-up data are available for 34 subjects, 7 of which were alive at last follow-up. Of these 7 patients, 5 had PFS at 12 weeks.

### Safety

All 44 patients on treatment were assessed for safety. A total of 769 AEs were reported and all patients reported at least one AE. The most frequent cabozantinib-related AEs reported in ≥ 10% of patients included fatigue, diarrhea, hypertension, nausea, decreased appetite, weight loss, proteinuria, constipation, palmar-plantar erythrodysaesthesia syndrome, abdominal pain, vomiting, thrombocytopenia, alanine aminotransferase increase, and hypothyroidism (Table 2). A total of 93 AEs were recorded as ≥ grade 3 which occurred in 34 subjects (77%). Serious AEs (SAE) were reported in 20 (45%) patients with a total of 37 documented SAEs and fourteen (38%) of SAEs were recorded as at least possibly related to cabozantinib. Of the five grade 5 AEs that were observed, four were documented as not related to treatment (three disease progression, one disseminated intravascular coagulation) and one was documented as possibly related to treatment attributed to bowel perforation requiring ICU level care in the setting of radiographic evidence of disease progression.

**TABLE 2:** AEs occurring in ≥ 10% of patients from start of treatment to 30 days after end of treatment

	Patients with AE *N* (%)	Patients with AEs Grade ≥ 3 *N* (%)
Clinical adverse event (AE)		
Fatigue	28 (64%)	3 (7%)
Diarrhea	23 (52%)	3 (7%)
Hypertension	18 (41%)	10 (23%)
Nausea	18 (41%)	0 (0%)
Decreased appetite	16 (36%)	1 (2%)
Weight loss	15 (34%)	1 (2%)
Proteinuria	14 (32%)	3 (7%)
Constipation	14 (32%)	1 (2%)
Palmar-plantar erythrodysthesesia	13 (30%)	3 (7%)
Abdominal pain	13 (30%)	1 (2%)
Vomiting	13 (30%)	1 (2%)
Thrombocytopenia	11 (25%)	2 (5%)
ALT elevation	10 (23%)	2 (5%)
Hypothyroidism	10 (23%)	0 (0%)
Back pain	9 (20%)	3 (7%)
Muscle spasms	9 (20%)	0 (0%)
AST elevation	9 (20%)	0 (0%)
Dysgeusia	9 (20%)	0 (0%)
Hypokalemia	8 (18%)	3 (7%)
Pain in extremity	8 (18%)	1 (2%)
Cough	8 (18%)	0 (0%)
Pyrexia	8 (18%)	0 (0%)
Bilirubin elevation	7 (16%)	2 (5%)
Alkaline phosphatase elevation	7 (16%)	2 (5%)
Anemia	7 (16%)	0 (0%)
Hematuria	7 (16%)	0 (0%)
Dizziness	7 (16%)	0 (0%)
GERD	7 (16%)	0 (0%)
Peripheral edema	6 (14%)	0 (0%)
Leukopenia	6 (14%)	0 (0%)
Hypomagnesemia	6 (14%)	0 (0%)
Peripheral neuropathy	6 (14%)	0 (0%)
Oropharyngeal pain	6 (14%)	0 (0%)
Dyspnea	5 (11%)	1 (2%)
Hypophosphatemia	5 (11%)	1 (2%)
Upper abdominal pain	5 (11%)	0 (0%)
Flatulence	5 (11%)	0
Pruritus	5 (11%)	0

Dose reduction of cabozantinib occurred in 24 (55%) patients and 5 (11%) patients were reduced to the lowest dose level of 20 mg/day. Two patients were discontinued on treatment due to toxicity.

### Association Between ctDNA Concentration Variation and Treatment Efficacy

The presence of pathologic mutations in study participants was determined using cfDNA extracted from whole blood. The association between mutation prevalence and response to therapy was analyzed in the patients for which a pretreatment and posttreatment sample was obtained, and in whom there was a greater than 20% change in mutation prevalence (*n* = 14). Of the 7 patients with SD or clinical response to therapy, 5 showed a decrease in the prevalence of known pathologic mutations, while 2 had an increase in mutation prevalence. In patients whose disease clinically progressed during the study, 5 showed increase pathologic mutation prevalence, while 2 had a decrease in pathologic mutation prevalence. While these results show association between clinical disease and mutational load, the association was not statistically significant (Fisher exact test *P* = 0.143).

## Discussion

We demonstrated that cabozantinib has clinical activity in patients with refractory, heavily pretreated mCRC. Of the 40 evaluable patients, 45% of patients (*n* = 18) met the 12-week PFS primary endpoint in a treatment refractory patient population with a median of 4 previous lines of treatment. The survival endpoints of metastatic PFS (mPFS) and median OS (mOS) were 3.0 months and 8.3 months, respectively. One patient with PR maintained response through study closure and despite dose reduction to 40 mg/day of cabozantinib. ctDNA analysis also showed a trend toward concordance between mutational load and clinical efficacy. The safety profile of cabozantinib is consistent with previous studies in other solid tumors with the most frequent AEs were fatigue (64%), diarrhea (52%), and hypertension (41%; refs. 20–22).

The patient demographics, tolerability of treatment, and efficacy of cabozantinib are comparable with the phase III CORRECT and RECOURSE trials that led to FDA approval of regorafenib and trifluridine/tipiracil (TAS-102), respectively, for treatment of refractory mCRC. In the CORRECT trial, the regorafenib group had a mPFS of 1.9 months and a mOS of 6.4 months (9). Similarly, the RECOURSE trial investigating TAS-102 showed a mPFS of 2.0 months and mOS of 7.1 months in the investigational arm (11). ORRs reported in these trials were 1% and 1.6%, respectively. Oral VEGFR TKI agents apatinib and fruquintinib have shown mPFS of approximately 4 months in Asian patients with refractory, mCRC (27, 28).

A trend toward improved survival of patients with *RAS* WT versus *RAS* MT tumors was also apparent in this study. A 2.2-month difference in mPFS and a 3.4-month difference in mOS was observed in patients with *RAS* WT versus *RAS* MT tumors (*P* = 0.16 and *P* = 0.13, respectively). Of note, correlation between *RAS* mutational status and treatment response has not been reported with apatinib or fruquitinib. Importantly, this study did not intend to formally evaluate *RAS* mutational status as a primary endpoint, and the differences between groups could be confounded by prognosis, tumor sidedness, treatment history, and other factors. Despite preclinical data suggesting a possible improved response to cabozantinib in *PIK3CA* MT colorectal cancer tumors, there was no observed survival difference in subgroup analysis using *PIK3CA* mutational status. No additional clinically relevant mutations including *HER2* overexpression, *BRAF* V600E mutation, *NTRK* fusions, and MSI-H tumor types were reported.

In this trial, we showed an association between reduction of ctDNA and patients who met the 12-week PFS mark. Although a limitation of our ctDNA analysis include small sample size, we believe our results are hypothesis generating as they are consistent with previous reports showing that ctDNA may offer early response analysis of treatment (29). In the metastatic setting, ctDNA may offer predictive and prognostic information although data from prospective trials are limited (30–32). Blood-based analyses to better characterize and quantify biomarkers in patients with heavily pretreated mCRC continues to evolve with the hope of offsetting the challenges of tumor heterogeneity, comorbidity, and costs inherent to tissue biopsies (33). Our understanding of how to best utilize ctDNA has been challenging due to mixed clinical results. For example, in a phase Ib study of 24 patients, evaluation of *MET* amplification in cfDNA as a predictive biomarker in patients with *MET*-amplified mCRC undergoing cabozantinib plus panitumuab showed response in only 1 of 5 patients (34, 35). Despite data showing the importance of aberrant MET expression in acquired therapeutic resistance in mCRC, the predictive value of MET expression by IHC has not been proven (36). While there are several blood-based assays commercially available to molecularly characterize tumors and monitor response via ctDNA, data on how to utilize these technologies as a predictive tool are limited (37). Large, prospective trials are needed to help establish the role for ctDNA testing as a predictive tool in the management of patients with mCRC.

Cabozantinib has also shown clinical activity in other trials investigating its use in mCRC. The EGFR antibodies panitumumab and cetuximab have been approved on the basis of incremental survival benefit in treatment of *RAS* WT mCRC (38–41). Analogous to antiangiogenic therapy in mCRC, acquired resistance to anti-*EGFR* therapies through aberrant *MET* overexpression has been reported previously (42–44). In a recently published phase Ib clinical trial of 25 patients with *RAS* WT, mCRC, the combination of cabozantinib plus panitumumab, a fully humanized mAb targeting EGFR, demonstrated an ORR of 16%, mPFS of 15.8 weeks, and mOS of 51.8 weeks (34). There were no reported grade 5 AEs, 5 patients discontinued treatment due to toxicity, and 72% of patients required a dose reduction of cabozantinib. A high degree of grade ≥ 3 AEs were observed, although these data were similar to safety outcomes noted in previously reported trials in similar patient populations. Tolerability did not appear to correlate with clinical response.

Immunomodulatory effects of cabozantinib via downregulation of myeloid-derived suppressor cells and regulatory T cells have also been reported previously (45, 46). The *Tyro3*, *AXL*, and *MerTK* (TAM) receptor family regulate tumor-associated macrophages that elicit an immunosuppressive tumor microenvironment, and blockade of TAM receptors via cabozantinib may invigorate antitumor immune activation (47, 48). Because of favorable immunomodulatory properties, cabozantinib in combination with immune checkpoint inhibitors has shown promise in a variety of solid tumor types and clinical settings including metastatic renal cell, hepatocellular carcinoma, and mCRC (49–52). In the phase Ib CAMILLA basket trial, the combination of cabozantinib plus durvalumab, an anti-programmed death-ligand1 (PD-L1) mAb, led to two PRs out of 13 treated patients with mCRC and an ORR of 21% (53). A phase I dose-expansion trial investigating this combination in mCRC is ongoing (NCT03539822). On the basis of preclinical data showing synergy between cabozantinib and checkpoint inhibitors in MSS colorectal cancer PDXs, a phase II trial investigating cabozantinib plus nivolumab, an anti-programmed death-1 (PD-1) mAb, in treatment of refractory mCRC is also ongoing (NCT03170960; ref. 54).

Clinical data using other multi-TKIs plus checkpoint inhibition has shown mixed results. The Japanese phase Ib REGONIVO trial investigating regorafenib plus nivolumab demonstrated an impressive ORR of 29% in patients with refractory, mismatch repair proficient mCRC (55). However, a follow-up North American phase II trial using regorafenib plus nivolumab combination showed an ORR of 7.1% with a mPFS of 2.0 months in patients with refractory mCRC. Subgroup analysis demonstrated higher response rates and survival in patients without liver metastases compared with patients with liver metastases (ORR of 21.7% vs. 0%, respectively). Whether cabozantinib plus immunotherapy will demonstrate improved outcomes in mCRC is eagerly awaited, and further insight into subgroup analysis through biomarker-driven assessment is needed.

There were several limitations to this trial including the open-label, nonrandomized study design. Without a control group, the interpretation of the observed PFS and survival endpoints become less clear as the outcomes may be influenced by a highly selected patient population with a more favorable prognosis. Also, the benefit of a surrogate endpoint of PFS rate at 12 weeks may not translate to an improved OS benefit, which is the gold standard for assessing efficacy of cancer therapeutics (56). In addition, we used investigator assessment of RECIST v1.1 for radiographic assessments, making investigator bias difficult to control. Finally, information regarding molecular profiling from tumor tissue was collected via historical records performed prior to study enrollment using various platforms based on investigator discretion.

We believe the results of our trial support further investigation of cabozantinib as a single agent and/or in combination in the treatment of mCRC. In addition, this study may provide a useful baseline in evaluating efficacy in combinational trials. On the basis of the number of dose reductions required and durable response observed at 40 mg daily dosing, future designs may consider investigating cabozantinib 40 mg orally daily dosage. Studies using cabozantinib plus checkpoint inhibitors in mCRC are currently underway and results are highly anticipated (NCT04963283; NCT03539822; NCT03170960). On the basis of subgroup analysis, patients who harbor a *RAS* WT tumor may derive greater benefit from cabozantinib treatment; however, this is strictly a hypothesis generating observation. Studies using ctDNA as a predictive biomarker may also improve upon assessment of treatment response. Future research should aim to identify and validate predictive biomarkers, refine ctDNA as a predictive tool for disease response, and will hopefully refine the patient population that may derive the most benefit from cabozantinib.

## Supplementary Material

Supplementary Figure 1 KM curves for PIK3CA mutant subjects.

Supplementary Figure 2 Waterfall plot for maximum treatment response.

## References

[1] Siegel RL , Miller KD, Sauer AG, Fedewa SA, Butterly LF, Anderson JC, . Colorectal cancer statistics. CA Cancer J Clin 2020;70:145–64. 32133645 10.3322/caac.21601

[2] National Comprehensive Cancer Network. NCCN clinical practice guidelines in oncology (NCCN guidelines): colon cancer. Version 3; 2021. Available from: https://www.nccn.org/professionals/physician_gls/pdf/colon.pdf.

[3] Kopetz S , Grothey A, Yaeger R, Van Cutsem E, Desai J, Yoshino T, . Encorafenib, binimetinib, and cetuximab in BRAF V600E–mutated colorectal cancer. N Engl J Med 2019;381:1632–43. 31566309 10.1056/NEJMoa1908075

[4] André T , Shiu K-K, Kim TW, Jensen BV, Jensen LH, Punt C, . Pembrolizumab in microsatellite-instability–high advanced colorectal cancer. N Engl J Med 2020;383:2207–18. 33264544 10.1056/NEJMoa2017699

[5] Lenz H-J , Lonardi S, Zagonel V, Cutsem EV, Limon ML, Wong KYM, . Nivolumab plus low-dose ipilimumab as first-line therapy in microsatellite instability-high/DNA mismatch repair deficient metastatic colorectal cancer: clinical update. J Clin Oncol 38:4s, 2020 (suppl; abstr 11).

[6] Ruiz-Bañobre J , Kandimalla R, Goel A. Predictive biomarkers in metastatic colorectal cancer: a systematic review. JCO Precis Oncol 2019;3:PO.18.00260. 32914007 10.1200/PO.18.00260 PMC7446314

[7] Ou SI , Jänne PA, Leal TA, Rybkin II, Sabari JK, Barve MA, . First-in-human phase I/IB dose-finding study of adagrasib (MRTX849) in patients with advanced KRAS(G12C) solid tumors (KRYSTAL-1). J Clin Oncol 2022:40:2530–8. 35167329 10.1200/JCO.21.02752 PMC9362872

[8] Hong DS , Fakih MG, Strickler JH, Desai J, Durm GA, Shapiro GI, . KRASG12C inhibition with sotorasib in advanced solid tumors. N Engl J Med 2020;383:1207–17. 32955176 10.1056/NEJMoa1917239 PMC7571518

[9] Grothey A , Van Cutsem E, Sobrero A, Siena S, Falcone A, Ychou M, . Regorafenib monotherapy for previously treated metastatic colorectal cancer (CORRECT): an international, multicentre, randomised, placebo-controlled, phase 3 trial. Lancet 2013;381:303–12. 23177514 10.1016/S0140-6736(12)61900-X

[10] Mayer RJ , Van Cutsem E, Falcone A, Yoshino T, Garcia-Carbonero R, Mizunuma N, . Randomized trial of TAS-102 for refractory metastatic colorectal cancer. N Engl J Med 2015;372:1909–19. 25970050 10.1056/NEJMoa1414325

[11] Ferrara N . The role of VEGF in the regulation of physiological and pathological angiogenesis. EXS 2005:209–31. 15617481 10.1007/3-7643-7311-3_15

[12] Hurwitz H , Fehrenbacher L, Novotny W, Cartwright T, Hainsworth J, Heim W, . Bevacizumab plus irinotecan, fluorouracil, and leucovorin for metastatic colorectal cancer. N Engl J Med 2004;350:2335–42. 15175435 10.1056/NEJMoa032691

[13] Van Cutsem E , Tabernero J, Lakomy R, Prenen H, Prausová J, Macarulla T, . Addition of aflibercept to fluorouracil, leucovorin, and irinotecan improves survival in a phase III randomized trial in patients with metastatic colorectal cancer previously treated with an oxaliplatin-based regimen. J Clin Oncol 2012;30:3499–506. 22949147 10.1200/JCO.2012.42.8201

[14] De Oliveira AT , Matos D, Logullo AF, DAS SR, Neto RA, Filho AL, . MET is highly expressed in advanced stages of colorectal cancer and indicates worse prognosis and mortality. Anticancer Res 2009;29:4807–11. 20032439

[15] Gherardi E , Birchmeier W, Birchmeier C., Vande woude G. targeting MET in cancer: rationale and progress. Nat Rev Cancer 2012;12:89–103. 22270953 10.1038/nrc3205

[16] Takeuchi H , Bilchik A, Saha S, Turner R, Wiese D, Tanaka M, . c-MET expression level in primary colon cancer: a predictor of tumor invasion and lymph node metastases. Clin Cancer Res 2003;9:1480–8. 12684423

[17] Rimassa L , Bozzarelli S, Pietrantonio F, Cordio S, Lonardi S, Toppo L, . Phase II study of tivantinib and cetuximab in patients with KRAS wild-type metastatic colorectal cancer with acquired resistance to EGFR inhibitors and emergence of MET overexpression: lesson learned for future trials with EGFR/MET dual inhibition. Clin Colorectal Cancer 2019;18:125–32. 30846365 10.1016/j.clcc.2019.02.004

[18] Liu Y , Yu XF, Zou J, Luo ZH. Prognostic value of c-Met in colorectal cancer: a meta-analysis. World J Gastroenterol 2015;21:3706–10. 25834339 10.3748/wjg.v21.i12.3706 PMC4375596

[19] Scott AJ , Arcaroli JJ, Bagby SM, Yahn R, Huber KM, Serkova NJ, . Cabozantinib exhibits potent antitumor activity in colorectal cancer patient-derived tumor xenograft models via autophagy and signaling mechanisms. Mol Cancer Ther 2018;17:2112–22. 30026382 10.1158/1535-7163.MCT-17-0131 PMC6168336

[20] Elisei R , Schlumberger MJ, Muller SP, Schoffski P, Brose MS, Shah MH, . Cabozantinib in progressive medullary thyroid cancer. J Clin Oncol 2013;31:3639–46. 24002501 10.1200/JCO.2012.48.4659 PMC4164813

[21] Choueiri TK , Escudier B, Powles T, Mainwaring PN, Rini BI, Donskov F, . Cabozantinib versus everolimus in advanced renal-cell carcinoma. N Engl J Med 2015;373:1814–23. 26406150 10.1056/NEJMoa1510016 PMC5024539

[22] Abou-Alfa GK , Meyer T, Cheng A-L, El-Khoueiry AB, Rimassa L, Ryoo B-Y, . Cabozantinib in patients with advanced and progressing hepatocellular carcinoma. N Engl J Med 2018;379:54–63. 29972759 10.1056/NEJMoa1717002 PMC7523244

[23] Schöffski P , Mir O, Kasper B, Papai Z, Blay J-Y, Italiano A, . Activity and safety of the multi-target tyrosine kinase inhibitor cabozantinib in patients with metastatic gastrointestinal stromal tumour after treatment with imatinib and sunitinib: European organisation for research and treatment of cancer phase II trial 1317 'CaboGIST'. Eur J Cancer 2020;134:62–74. 32470848 10.1016/j.ejca.2020.04.021

[24] Song EK , Tai WM, Messersmith WA, Bagby S, Purkey A, Quackenbush KS, . Potent antitumor activity of cabozantinib, a c-MET and VEGFR2 inhibitor, in a colorectal cancer patient-derived tumor explant model. Int J Cancer 2015;136:1967–75. 25242168 10.1002/ijc.29225 PMC4323738

[25] Smith M , Bono JD, Sternberg C, Moulec SL, Oudard S, Giorgi UD, . Phase III study of cabozantinib in previously treated metastatic castration-resistant prostate cancer: COMET-1. J Clin Oncol 2016;34:3005–13. 27400947 10.1200/JCO.2015.65.5597

[26] Choueiri TK , Escudier B, Powles T, Mainwaring PN, Rini BI, Donskov F, . Cabozantinib versus everolimus in advanced renal-cell carcinoma. N Engl J Med 2015;373:1814–23. 26406150 10.1056/NEJMoa1510016 PMC5024539

[27] Wang F , Yuan X, Jia J, Bi X, Zhou Z, Zhou Q, . Apatinib monotherapy for chemotherapy-refractory metastatic colorectal cancer: a multi-centre, single-arm, prospective study. Sci Rep 2020;10:6058. 32269247 10.1038/s41598-020-62961-5 PMC7142071

[28] Dasari A , Yao JC, Sobrero AF, Yoshino T, Schelman WR, Nanda S, . FRESCO-2: a global phase III study of the efficacy and safety of fruquintinib in patients (pts) with metastatic colorectal cancer (mCRC). J Clin Oncol 39:3s, 2021 (suppl; abstr TPS154). 10.2217/fon-2021-0202 33993740

[29] Garlan F , Laurent-Puig P, Sefrioui D, Siauve N, Didelot A, Sarafan-Vasseur N, . Early evaluation of circulating tumor DNA as marker of therapeutic efficacy in metastatic colorectal cancer patients (PLACOL Study). Clin Cancer Res 2017;23:5416–25. 28576867 10.1158/1078-0432.CCR-16-3155

[30] Dasari A , Morris VK, Allegra CJ, Atreya C, Benson AB, Boland P, . ctDNA applications and integration in colorectal cancer: an NCI colon and rectal–anal task forces whitepaper. Nat Rev Clin Oncol 2020;17:757–70. 32632268 10.1038/s41571-020-0392-0 PMC7790747

[31] Parikh AR , Van Seventer EE, Siravegna G, Hartwig AV, Jaimovich A, He Y, . Minimal residual disease detection using a plasma-only circulating tumor DNA assay in patients with colorectal cancer. Clin Cancer Res 2021;27:5586–94. 33926918 10.1158/1078-0432.CCR-21-0410 PMC8530842

[32] Morris VK , Raghav KPS, Dasari A, Overman MJ, Kee BK, Johnson B, . Utility of circulating tumor DNA in the clinical management of patients with BRAFV600E metastatic colorectal cancer. J Clin Oncol 39:3s, 2021 (suppl; abstr 119).

[33] Strickler JH , Loree JM, Ahronian LG, Parikh AR, Niedzwiecki D, Pereira AAL, . Genomic landscape of cell-free DNA in patients with colorectal cancer. Cancer Discov 2018;8:164–73. 29196463 10.1158/2159-8290.CD-17-1009 PMC5809260

[34] Strickler JH , Rushing CN, Uronis HE, Morse MA, Niedzwiecki D, Blobe GC, . Cabozantinib and panitumumab for RAS wild-type metastatic colorectal cancer. Oncologist 2021;26:465–e917. 33469991 10.1002/onco.13678 PMC8176979

[35] Jia J , Morse MA, Nagy RJ, Lanman RB, Strickler JH. Cell-free DNA profiling to discover mechanisms of exceptional response to cabozantinib plus panitumumab in a patient with treatment refractory metastatic colorectal cancer. Front Oncol 2018;8:305. 30211110 10.3389/fonc.2018.00305 PMC6121109

[36] Bendell JC , Hochster H, Hart LL, Firdaus I, Mace JR, McFarlane JJ, . A phase II randomized trial (GO27827) of first-line FOLFOX plus bevacizumab with or without the MET inhibitor onartuzumab in patients with metastatic colorectal cancer. Oncologist 2017;22:264–71. 28209746 10.1634/theoncologist.2016-0223 PMC5344636

[37] Lanman RB , Mortimer SA, Zill OA, Sebisanovic D, Lopez R, Blau S, . Analytical and clinical validation of a digital sequencing panel for quantitative, highly accurate evaluation of cell-free circulating tumor DNA. PLoS One 2015;10:e0140712. 26474073 10.1371/journal.pone.0140712 PMC4608804

[38] Douillard JY , Oliner KS, Siena S, Tabernero J, Burkes R, Barugel M, . Panitumumab-FOLFOX4 treatment and RAS mutations in colorectal cancer. N Engl J Med 2013;369:1023–34. 24024839 10.1056/NEJMoa1305275

[39] Douillard JY , Siena S, Cassidy J, Tabernero J, Burkes R, Barugel M, . Final results from PRIME: randomized phase III study of panitumumab with FOLFOX4 for first-line treatment of metastatic colorectal cancer. Ann Oncol 2014;25:1346–55. 24718886 10.1093/annonc/mdu141

[40] Amado RG , Wolf M, Peeters M, Van Cutsem E, Siena S, Freeman DJ, . Wild-type KRAS is required for panitumumab efficacy in patients with metastatic colorectal cancer. J Clin Oncol 2008;26:1626–34. 18316791 10.1200/JCO.2007.14.7116

[41] Heinemann V , von Weikersthal LF, Decker T, Kiani A, Vehling-Kaiser U, Al-Batran SE, . FOLFIRI plus cetuximab versus FOLFIRI plus bevacizumab as first-line treatment for patients with metastatic colorectal cancer (FIRE-3): a randomised, open-label, phase 3 trial. Lancet Oncol 2014;15:1065–75. 25088940 10.1016/S1470-2045(14)70330-4

[42] Raghav K , Morris V, Tang C, Morelli P, Amin HM, Chen K, . MET amplification in metastatic colorectal cancer: an acquired response to EGFR inhibition, not a *de novo* phenomenon. Oncotarget 2016;7:54627–31. 27421137 10.18632/oncotarget.10559 PMC5342368

[43] Bardelli A , Corso S, Bertotti A, Hobor S, Valtorta E, Siravegna G, . Amplification of the MET receptor drives resistance to anti-EGFR therapies in colorectal cancer. Cancer Discov 2013;3:658–73. 23729478 10.1158/2159-8290.CD-12-0558 PMC4078408

[44] Pietrantonio F , Vernieri C, Siravegna G, Mennitto A, Berenato R, Perrone F, . Heterogeneity of acquired resistance to anti-EGFR monoclonal antibodies in patients with metastatic colorectal cancer. Clin Cancer Res 2017;23:2414–22. 27780856 10.1158/1078-0432.CCR-16-1863

[45] Apolo AB , Tomita Y, Lee M-J, Lee S, Frosch A, Steinberg SM, . Effect of cabozantinib on immunosuppressive subsets in metastatic urothelial carcinoma. J Clin Oncol 32:15s, 2014 (suppl; abstr 4501).

[46] Holokai L , Chakrabarti J, Lundy J, Croagh D, Adhikary P, Richards SS, . Murine- and human-derived autologous organoid/immune cell co-cultures as pre-clinical models of pancreatic ductal adenocarcinoma. Cancers 2020;12:3816.. 33348809 10.3390/cancers12123816 PMC7766822

[47] Myers KV , Amend SR, Pienta KJ. Targeting Tyro3, Axl and MerTK (TAM receptors): implications for macrophages in the tumor microenvironment. Mol Cancer 2019;18:94. 31088471 10.1186/s12943-019-1022-2 PMC6515593

[48] Hara T , Kimura A, Miyazaki T, Tanaka H, Morimoto M, Nakai K, . Cabozantinib inhibits AXL- and MET-dependent cancer cell migration induced by growth-arrest-specific 6 and hepatocyte growth factor. Biochem Biophys Rep 2020;21:100726. 32055714 10.1016/j.bbrep.2020.100726 PMC7005370

[49] Bergerot P , Lamb P, Wang E, Pal SK. Cabozantinib in combination with immunotherapy for advanced renal cell carcinoma and urothelial carcinoma: rationale and clinical evidence. Mol Cancer Ther 2019;18:2185–93. 31792125 10.1158/1535-7163.MCT-18-1399

[50] Choueiri TK , Powles T, Burotto M, Escudier B, Bourlon MT, Zurawski B, . Nivolumab plus cabozantinib versus sunitinib for advanced renal-cell carcinoma. N Engl J Med 2021;384:829–41. 33657295 10.1056/NEJMoa2026982 PMC8436591

[51] Ho WJ , Zhu Q, Durham J, Popovic A, Xavier S, Leatherman J, . Neoadjuvant cabozantinib and nivolumab convert locally advanced hepatocellular carcinoma into resectable disease with enhanced antitumor immunity. Nat Cancer 2021;2:891–903. 34796337 10.1038/s43018-021-00234-4 PMC8594857

[52] Yau T , Zagonel V, Santoro A, Acosta-Rivera M, Choo SP, Matilla A, . Nivolumab (NIVO) + ipilimumab (IPI) + cabozantinib (CABO) combination therapy in patients (pts) with advanced hepatocellular carcinoma (aHCC): results from CheckMate 040. J Clin Oncol 38:4s, 2020 (suppl;abstr 478).

[53] Saeed A , Phadnis M, Park R, Sun W, RMdT A-R, Baranda JC, . Cabozantinib (cabo) combined with durvalumab (durva) in gastroesophageal (GE) cancer and other gastrointestinal (GI) malignancies: preliminary phase Ib CAMILLA study results. J Clin Oncol 38:15s, 2020 (suppl; abstr 4563).

[54] Lang J , Leal AD, Hartmann SJ, Marin-Jimenez J, Shulman J, Capasso A, . Cabozantinib sensitizes microsatellite stable colorectal cancer to immune checkpoint inhibition by immune modulation in humanized mouse models [abstract]. In: Proceedings of the AACR Special Conference on Tumor Immunology and Immunotherapy; 2019 Nov 17–20; Boston, MA. Philadelphia (PA): AACR; Cancer Immunol Res 2020;8(3 Suppl):Abstract nr A109.

[55] Hara H , Fukuoka S, Takahashi N, Kojima T, Kawazoe A, Asayama M, . Regorafenib plus nivolumab in patients with advanced colorectal or gastric cancer: an open-label, dose-finding, and dose-expansion phase 1b trial (REGONIVO, EPOC1603). Ann Oncol 2019;30:iv124. 10.1200/JCO.19.03296 32343640

[56] Wilson MK , Karakasis K, Oza AM. Outcomes and endpoints in trials of cancer treatment: the past, present, and future. Lancet Oncol 2015;16:e32–42. 25638553 10.1016/S1470-2045(14)70375-4

