# Supplementary material for: A Phase II Study Investigating Cabozantinib in Patients with Refractory Metastatic Colorectal Cancer (AGICC 17CRC01)
Source: Cancer Res Commun. 2022 Oct 14;2(10):1188–96. doi: 10.1158/2767-9764.CRC-22-0169 (PMC10035393; doi:10.1158/2767-9764.CRC-22-0169)
Supplement: Supplementary Figure 1 — KM curves for PIK3CA mutant subjects. [file crc-22-0169-s01.pptx]

## Slide 1
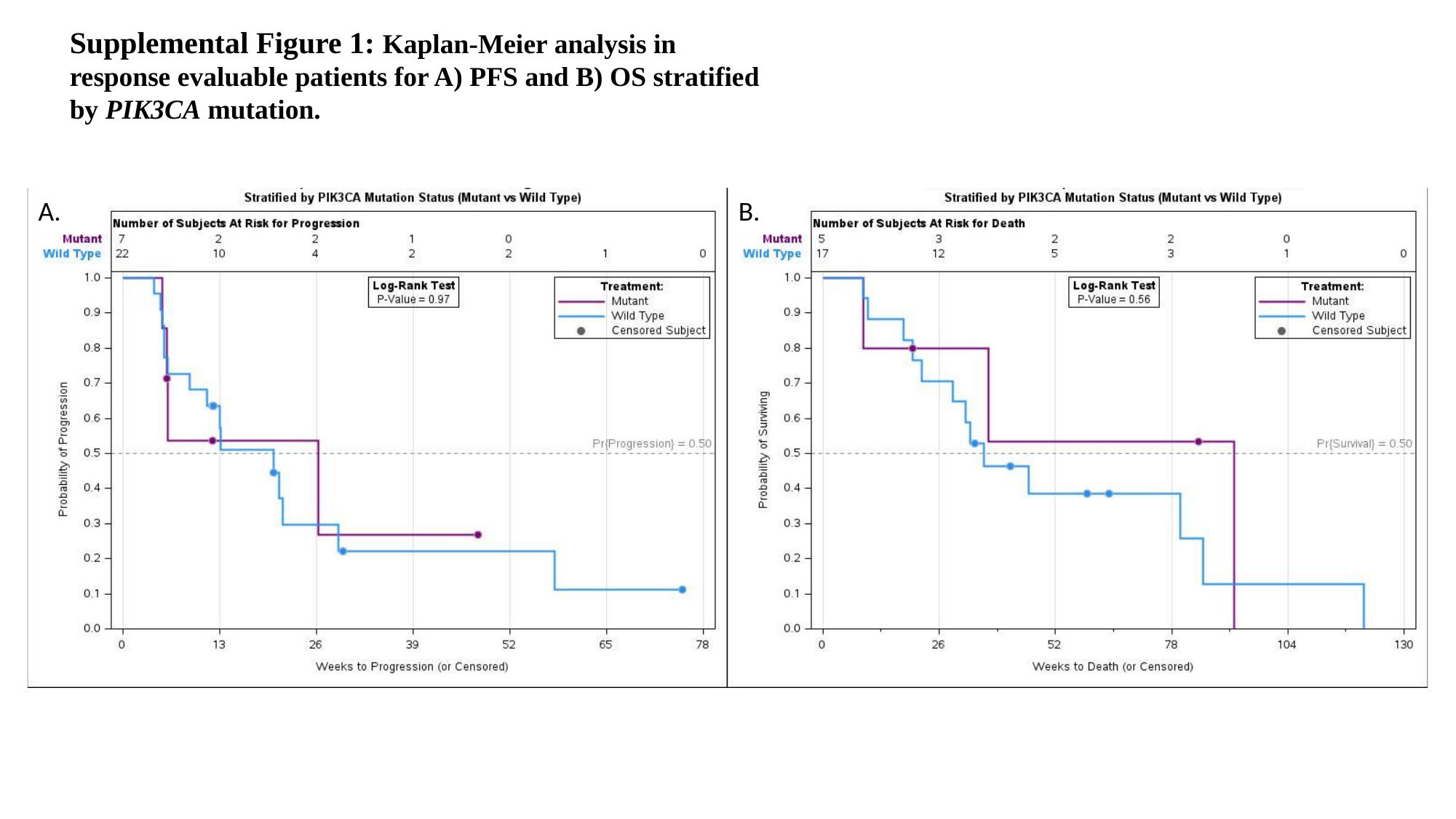

Supplemental Figure 1: Kaplan-Meier analysis in response evaluable patients for A) PFS and B) OS stratified by PIK3CA mutation.
A.
B.
