# Supplementary material for: A Phase II Study Investigating Cabozantinib in Patients with Refractory Metastatic Colorectal Cancer (AGICC 17CRC01)
Source: Cancer Res Commun. 2022 Oct 14;2(10):1188–96. doi: 10.1158/2767-9764.CRC-22-0169 (PMC10035393; doi:10.1158/2767-9764.CRC-22-0169)
Supplement: Supplementary Figure 2 — Waterfall plot for maximum treatment response. [file crc-22-0169-s02.pptx]

## Slide 1
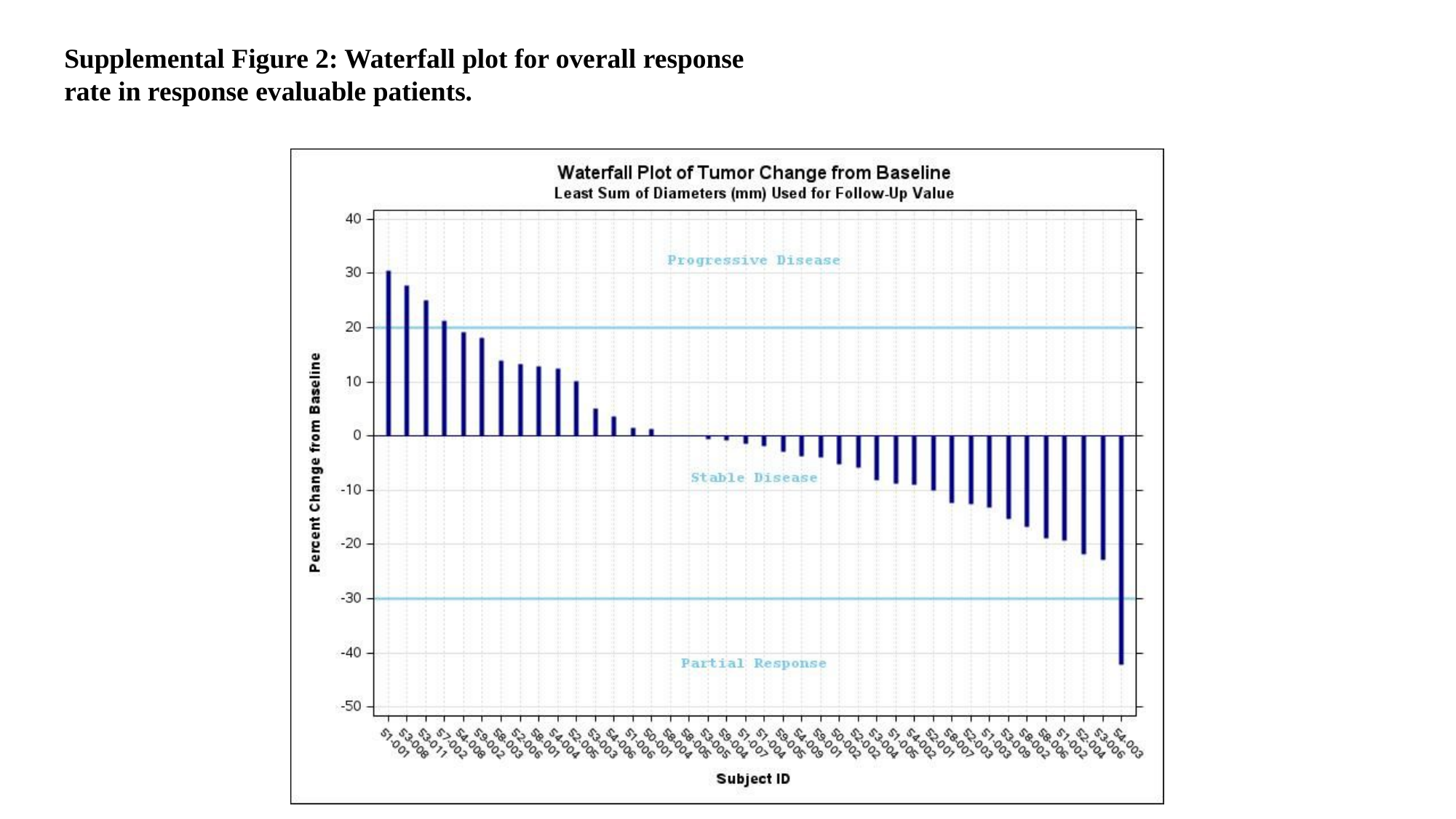

Supplemental Figure 2: Waterfall plot for overall response rate in response evaluable patients.
